# HE-BiDet: A Hardware Efficient Binary Neural Network Accelerator for Object Detection in SAR Images

**DOI:** 10.3390/mi16050549

**Published:** 2025-04-30

**Authors:** Dezheng Zhang, Zehan Liang, Rui Cen, Zhihong Yan, Rui Wan, Dong Wang

**Affiliations:** 1 Institute of Information Science, Beijing Jiaotong University, Beijing 100044, China; dezhengzhang@bjtu.edu.cn (D.Z.); 23120297@bjtu.edu.cn (Z.L.); 22120306@bjtu.edu.cn (R.C.); 23115053@bjtu.edu.cn (Z.Y.); 22110093@bjtu.edu.cn (R.W.); 2 Beijing Key Laboratory of Advanced Information Science and Network Technology, Beijing 100044, China

**Keywords:** binary neural networks, ship detection, synthetic aperture radar (SAR), field programmable gate array (FPGA)

## Abstract

Convolutional Neural Network (CNN)-based Synthetic Aperture Radar (SAR) target detection eliminates manual feature engineering and improves robustness but suffers from high computational costs, hindering on-satellite deployment. To address this, we propose HE-BiDet, an ultra-lightweight Binary Neural Network (BNN) framework co-designed with hardware acceleration. First, we develop an ultra-lightweight SAR ship detection model. Second, we design a BNN accelerator leveraging four-directions of parallelism and an on-chip data buffer with optimized addressing to feed the computing array efficiently. To accelerate post-processing, we introduce a hardware-based threshold filter to eliminate redundant anchor boxes early and a dedicated Non-Maximum Suppression (NMS) unit. Evaluated on SAR-Ship, AirSAR-Ship 2.0, and SSDD, our model achieves 91.3%, 71.0%, and 92.7% accuracy, respectively. Implemented on a Xilinx Virtex-XC7VX690T FPGA, the system achieves 189.3 FPS, demonstrating real-time capability for spaceborne deployment.

## 1. Introduction

Taking advantage of the all-weather, all-day and high-resolution characteristics of spaceborne Synthetic Aperture Radar (SAR) imaging, along with advancements in Convolutional Neural Networks (CNNs), significant progress has been made in ship target detection. Ship detection based on SAR images is of significant importance in both civilian [1] and defense [2] applications. CNN-based ship detection methods can automatically learn complex features from large datasets, demonstrating the strong ability of generalization. Compared to traditional approaches, CNN-based methods particularly improve the robustness of ship detection in complex scenes [3], improve multi-scale ship detection [4,5], and offer advantages in the detection of small ship targets [6].

However, existing CNN-based ship detection models typically involve high computational and parameter complexity, making them only suitable for deployment on expensive Graphic Processing Units (GPUs) with high computational power and high power supplies. For example, the NanoDet model [7] achieved an accuracy of 92.5% and a detection speed of 191.5 FPS on the SSDD data set using an NVIDIA 2080 Ti GPU whose peak power consumption reaches 250 W. Similarly, the BANet [4] achieved 95.0% accuracy and 14 FPS detection speed in the HRSID data set using an NVIDIA P100 GPU whose peak power consumption reaches 300 W [8]. Moreover, due to the significant latency in satellite-to-ground communication and susceptibility to uncontrollable factors such as climate conditions and complex electromagnetic propagation environments, real-time target detection is often desired. In summary, designing a lightweight ship detection algorithm along with a computing platform deployable on satellites is of great importance. Spaceborne ship detection based on low-cost high-power-efficiency edge devices can significantly reduce inference latency and minimize communication overhead between satellites and the ground.

## 2. Related Work and Contributions

Due to its flexible and reconfigurable nature, FPGA can achieve efficient neural network inference through hardware–software co-design, making it an ideal choice for implementing SAR target detection algorithms in space environments. However, existing FPGA-based SAR image target detection systems still face numerous challenges. One of the primary issues is the difficulty in balancing algorithmic detection accuracy and hardware deployment efficiency. For example, the work in [9] first applies pseudo-color processing to SAR images to enhance ship detection capabilities, then develops the YOLO v5 model on a Xilinx XCVU9P FPGA device for ship detection. A total of 114.2 MB model parameters are stored in the on-chip URAMs, and activations are stored in the Block RAMs (BRAMs). This approach achieves a mean Average Precision (mAP) of 78.74% and a processing speed of 25.8 FPS on the SAR-Ship dataset [10]. However, this implementation consumes up to 406 URAMs, 1541 BRAMs, and 5107 DSP resources, significantly increasing the complexity of device selection and leading to higher overall system power consumption. In [11], an onboard ship detection system is proposed based on hardware–software co-design. This work introduces a hardware-guided structured pruning strategy to reduce computational and parameter complexity. Additionally, all convolutional layers of CNN are mapped onto the FPGA, forming a deeply pipelined neural network accelerator. That work also utilizes depthwise convolution to reduce the computational workload to further accelerate the inference. However, as noted in many previous studies [12,13], depthwise separable convolution differs from standard convolution in its computational pattern, posing significant challenges for hardware design. Moreover, due to its lower computational intensity, the performance is often limited by memory bandwidth [14,15].

The proposed system, implemented on a XC7VX690T FPGA, achieves an mAP of 93.3% and a detection frame rate of 636 FPS on the SSDD dataset. The high FPS is achieved with a large batch size of 50, which means that the end-to-end latency is about 76.68 ms. However, in space- or air-borne edge computing scenarios, the detection latency has more priority.

Additionally, because of the high resolution of SAR input images, a large number of candidate bounding boxes are generated. Then, post-processing is carried out to decode the coordinate and classification information and filter out the redundant candidate boxes. However, because of the low performance of the spaceborne processors, the post-processing stage is time-consuming. Although some studies [16,17] have explored accelerating the post-processing stage, their hardware consumption remains excessively high. For example, the work in [16] proposes an ultra-low latency NMS unit handling up to 65 candidate boxes, each with a dedicated IoU computation and storage unit. These units operate in a deeply pipelined structure, enabling efficient parallel processing. Each new bounding box is sequentially compared with stored candidates, and based on IoU and confidence score, the units decide whether to update existing boxes. That work achieves an end-to-end detection latency of only 2.13 ms. However, it leads to excessive resource consumption, including 5129 DSPs, 575.3 k logic elements and 7659 BRAM blocks. In particular, the post-processing unit alone consumes 695 DSPs, 425 BRAM blocks, and 86.6 k logic resources.

To address the above-mentioned challenges, in this work, we propose an ultra light-weight algorithm/hardware co-design SAR image target detection system. To the best of our knowledge, our design is the first BNN-based ship detection framework whose accuracy is comparable to that of traditional floating-point CNN models and can be efficiently deployed on edge-end FPGA devices with extremely low detection latency. Specifically, the main innovative points of this work are as follows:

(1) We propose an ultra light-weight BNN model, namely HE-BiDet, to carry out the ship detection task on the SAR imagery with low computation complexity. Both activation and weights are represented by 1-bit data, significantly reducing computational and storage overhead. With extreme binary quantization, feature pyramid structure, and model capacity enhancement, our model achieves a detection accuracy comparable to State-of-the-Art floating point ship detection models, while reducing the model size by a factor up to 18.9×.
(2) We design a low latency BNN inference accelerator for ship detection on SAR imagery. It utilizes four degrees of parallelism within the convolution inference calculation to achieve low detection latency. In addition, a novel on-chip data buffer and the corresponding data addressing algorithm are proposed to efficiently supply data to the computing array with high parallelism.
(3) We propose a “threshold first” post-processing unit to accelerate post-processing with low hardware consumption. This is based on our observation in the SSDD dataset that only 1.47% of the candidate boxes have confidence scores higher than the commonly used threshold of 0.01 [18]. Before confidence and coordinate decode, a parallel threshold hardware unit filters out redundant anchor boxes according to the raw data of the convolution output, which reduces the workload of subsequent operations. Therefore, subsequent decoding and NMS operations are performed with low latency even with low hardware consumption.
(4) Our SAR image target detection system is validated on a platform using the XC7VX690T FPGA device. The design achieves an mAP of 92.7%, an FPS of 80.5, and a latency of 12.4 ms on the SSDD data set. For the SAR-SHIP dataset, it achieves an mAP of 90.12%, an FPS of 189.3, and a latency of 5.2 ms. Compared with State-of-the-Art ship detection works on SAR imagery, our work achieves up to 6.34 times latency reduction and 15.8 times DSP consumption reduction.

## 3. BNN-Based Ship Detection Model

### 3.1. Network Structure Overview

**Figure 1:**
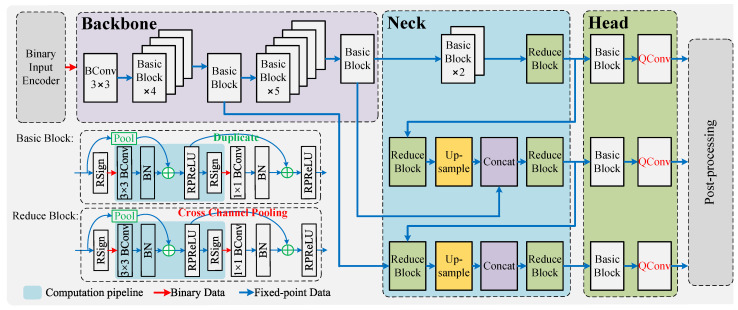
The network architecture of the proposed HE-BiDet.

The overall architecture of the ultra-lightweight and hardware-friendly SAR image target detection framework is shown in Figure 1. The proposed network is inspired by the YOLO series algorithm and consists of five main components: input encoding, backbone, neck, head, and post-processing. The input encoding unit extends the single-channel grayscale image into a 16-channel binary activation representation [19], enhancing feature expressiveness while maintaining computational efficiency. The backbone network extracts multi-scale features from different layers. The shallow layers focus on texture details, while the deeper layers focus on semantic information, enabling robust feature extraction across scales. The detection neck further refines these features by integrating information from multiple scales. It merges high-level semantic features with low-level texture details using a feature pyramid structure to enhance the ability to detect ships of varying sizes, which is an essential requirement for SAR-based maritime surveillance. The detection head employs binary and fixed-point convolutions to predict the location and classifications of bounding boxes efficiently. Finally, the post-processing unit decodes the coordinates and classification scores for candidate boxes, removes redundant detections using NMS, and generates the final detection results.

### 3.2. Basic Block Design

As shown in Figure 1, the proposed HE-BiDet model is mainly composed of basic blocks and reduce blocks, where the Binary Multiply Accumulate (BMAC) operation undertakes the major information extraction workload. Only the last three layers use 8-bit fixed-point convolution to extract the coordinate and confidence information. Moreover, the computational workload of fixed-point convolution is limited to a low level, which does not significantly increase the overall computational burden.

Basic blocks and reduce blocks are optimized on the bias of ReActNet [20]. The convolution inputs and weights are 1-bit data calculated by

(1) $$x^{b}=\mathrm{Sign}\left(x^{r}\right)=\left\{\begin{array}{c} +1,x^{r}>0\\ -1,x^{r}\leq 0 \end{array},\right.$$

where $x^{b}$ and $x^{r}$ denote the binary data and their corresponding real values. Each activation pixel can only represent −1 or 1, so the capacity of the model is greatly reduced.

To enhance the representational capacity of the network and improve the gradient optimization flow during backpropagation, real-valued shortcuts are adopted in the basic block designs. By incorporating the shortcut, the network can directly propagate the output of the previous layer to the next layer, which enhances the network’s expressive capacity. After introducing the shortcut operation, the forward propagation process of a binary convolution is denoted as

(2) $$A_{r}^{l+1}=\mathrm{Conv}\left[\mathrm{Sign}\left(A_{r}^{l}\right)\right]+A_{r}^{l}.$$

Moreover, in backward propagation, it facilitates a more efficient gradient flow and mitigates the gradient vanishing problem caused by binarization in traditional BNNs. The partial derivative during backpropagation is computed using the chain rule, i.e.,

(3) $$\frac{\partial A_{r}^{l+1}}{\partial A_{r}^{l}}=1+\frac{\partial A_{r}^{l+1}}{\partial \mathrm{Conv}}\;\cdot \;\frac{\partial \mathrm{Conv}}{\partial \mathrm{Sign}(A_{r}^{l})}\;\cdot \;\frac{\partial \mathrm{Sign}(A_{r}^{l})}{\partial A_{r}^{l}},$$

where, the first term 1 originates from the shortcut connection, indicating that the gradient is directly propagated through the shortcut path, thereby avoiding training issues caused by gradient vanishing. The rest represents the partial derivative corresponding to the binary convolutional operations. Through this mechanism, gradients are determined not only by the convolutional path but also by the shortcut path, which effectively strengthens the gradient flow and enables better parameter updates during network training.

### 3.3. Cross-Channel Pooling

In the basic block of ReActNet, due to the limitation of shortcut operation, the output channel number must be equal to one or two times the input channel number. This constrains the flexibility of the model structure, especially for the neck and head, where there are a lot of concatenation operations.

**Figure 2:**
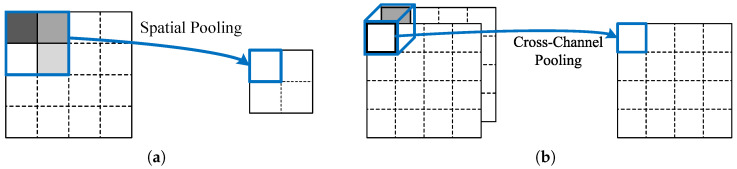
Illustration of spatial pooling and cross-channel pooling [24]: (**a**) Spatial pooling. (**b**) Cross-channel pooling.

To address these issues, inspired by the works in [21,22,23,24], we introduce inter-channel pooling operations in the convolution blocks of the neck network. The schematic diagrams of spatial pooling and inter-channel pooling are shown in Figure 2. Condensation-Net [24] reduces off-chip memory access by employing inter-channel pooling. PokeBNN [23] also introduce the inter-channel pooling, aiming at improving the network capacity of the BNN and improve the gradient backward flow to improve the performance of the model.

The inter-channel pooling enables implementing shortcut operations on activations with different numbers of channel, such as the reduce blocks in the neck of the HE-BiDet model. Therefore, on the one hand, the representational capacity of the binary neural network is enhanced; on the other hand, it also mitigates the potential gradient vanishing problem. The forward propagation process with inter-channel pooling is denoted as

(4) $$A_{r}^{l+1}=\mathrm{Conv}\left[\mathrm{Sign}\left(A_{r}^{l}\right)\right]+{\mathrm{Pool}}_{ch}\left(A_{r}^{l}\right),$$

where ${\mathrm{Pool}}_{ch}\left(X\right)$ denotes inter-channel pooling on matrix X. Compressing the feature map channels by a factor of *K* can be expressed as:

(5) $${\mathrm{Pool}}_{ch}{\left(X\right)}_{i}=\frac{1}{K}\sum_{0\leq k<K}X_{i\;\cdot \;K+k}$$

The gradient during backpropagation is given by:

(6) $$\frac{\partial A_{r}^{l+1}}{\partial A_{r}^{l}}=\frac{1}{K}+\frac{\partial A_{r}^{l+1}}{\partial \mathrm{Conv}}\;\cdot \;\frac{\partial \mathrm{Conv}}{\partial \mathrm{Sign}(A_{r}^{l})}\;\cdot \;\frac{\partial \mathrm{Sign}(A_{r}^{l})}{\partial A_{r}^{l}}$$

where the constant term $\frac{1}{K}$ is introduced due to the inter-channel pooling. This constant term helps with the back propagation of gradient.

## 4. Hardware Accelerator Design

**Figure 3:**
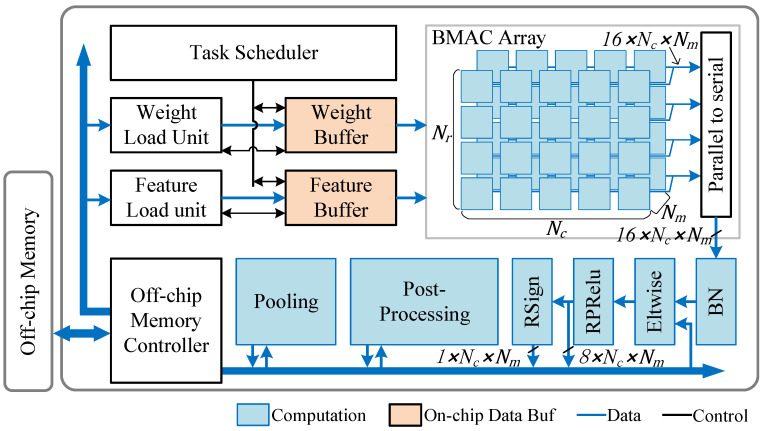
The architecture of the proposed BNN accelerator.

Object detection, as a core task in computer vision, often faces challenges such as high computational complexity and substantial memory access demands due to high resolution inputs. The architecture of the proposed BNN-based object detection accelerator is shown in Figure 3, which consists of data access units, on-chip buffers, binary multiply accumulation, and post processing units. The BMAC array accelerates the computation by utilizing four dimensions of parallelism within the convolution computation. Compared to the BNN accelerator [25] we previously proposed for classification tasks, this work proposes a novel on-chip data address mapping scheme and designs the corresponding memory access hardware units to meet the high-parallelism and low latency data access requirements. This approach improves off-chip memory efficiency with reduced hardware resource consumption. Addressing the need for higher convolution data bit-width in the object detection head to achieve precise localization of target positions, this chapter extends the configurable dual-function unit from [25] to design a multifunctional computing unit capable of performing convolution, linear transformation, and activation operations. Detailed computation mappings are provided for the convolution and Parametric ReLU (PReLU) operations. To address the issues of a large number of candidate boxes and the long post-processing latency caused by the large input size and high resolution of SAR images, a threshold unit and an NMS acceleration unit are proposed. Compared to the serial NMS algorithm in [26] and the fully parallel pipelined NMS algorithm in [16,17], this design strikes a balance between computational latency and resource consumption. Additionally, it allows for adjustable parallelism to adapt to different hardware platforms.

### 4.1. High Parallel Data Access System

The object detection task is characterized by high input image resolution and large data access volume, so, the efficiency of off-chip memory access significantly impacts the accelerator’s performance. Additionally, providing activations for high-parallelism convolution computing units is also a challenge. To address these two challenges, we first proposed an activation storage format for off-chip memory and its corresponding data access unit to fully utilize off-chip memory bandwidth resources. Furthermore, we also present an on-chip data buffer unit that supports multi-dimensional memory access, along with a corresponding data address mapping algorithm.

**Figure 4:**
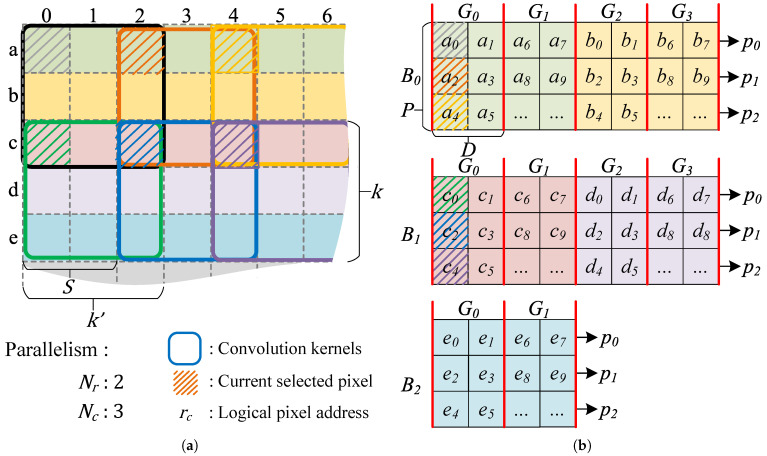
Feature map data prefetching strategy and the data addressing scheme: (**a**) Parallel convolution calculation is carried on the row and column directions, respectively. (**b**) The data address mapping that supports multi-dimensional data access in parallel. The rows of different input feature maps are highlighted in different colors.

**Multi-Dimensional Data Access Buffer Design.** Considering the parallelism of $N_{r}$ in the vertical direction and $N_{c}$ in the horizontal direction during convolution operations, a key challenge in the design is how to provide input activations in parallel to multiple convolution kernels under the constraint of limited on-chip buffer port width. To address the multi-dimensional data access requirements of the computing units, this section proposes an on-chip buffer design capable of parallel data access in both horizontal and vertical directions. For simplicity, a 2D convolution example is used as an illustration. For example, in Figure 4, the vertical parallelism equals to $N_{r}=2$ and the horizontal parallelism equals to $N_{c}=3$. Figure 4a illustrates the parallel convolution of the convolution window on the input feature map, while Figure 4b depicts the corresponding storage scheme of the input feature map in the on-chip memory.

In the vertical direction, as shown in Figure 4a, to meet the data requirements of $N_{r}$ convolution kernels along the height direction, the height of the prefetch window $H_{win}$ must satisfy:

(7) $$H_{win}=\left(N_{r}-1\right)\times S+K$$

Furthermore, as illustrated in Figure 4a, the data interval required by adjacent convolution kernels equals the convolution stride *S*. For example, the activation $a_{0}$ and $c_{0}$ are simultaneously fed into two adjacent convolution kernels along the height direction. As shown in Figure 4b, to support the parallel read requirement of the $N_{r}$ convolution kernels, the on-chip activation buffer is divided into multiple banks, each capable of storing up to *S* rows of input feature map data, and the number of banks is equal to $\left\lceil H_{win}/S\right\rceil$. This design avoids memory access conflicts caused by parallel data reads.

In the horizontal direction, data must be supplied simultaneously to the $N_{c}$ convolution kernels in parallel. Therefore, *P* ports are designed for each memory block, as shown in Figure 4b from $p_{0}$ to $p_{2}$. A single port can complete one data access within one clock cycle. Thus, to satisfy the simultaneous operation of the $N_{c}$ convolution kernels in the horizontal direction, the condition $P\geq N_{c}$ must be met.

Furthermore, to ensure that each port can only perform one data access per clock cycle, the length of continuously stored data *D* within each port should be less than or equal to the convolution stride *S*, that is, D≤S. For example, Figure 4b illustrates the case where D=2. Here, $a_{0}$ and $a_{1}$ are stored in port $p_{0}$, while $a_{2}$ is stored in port $p_{1}$. This arrangement ensures that no memory access conflict occurs when simultaneously accessing $a_{0}$ and $a_{2}$.

Finally, the range of address indexes required by the $N_{c}$ convolution kernels is $\left[x,x+\left(N_{c}-1\right)\times S\right]$, where *x* is the offset of the convolution sliding window. The data index range that can be accommodated by the *P* ports is [x,x+(P−1)×D]. To ensure that the memory blocks with *P* ports cover the input data required by the convolution kernels, the condition $\left(P-1\right)\times D\geq \left(N_{c}-1\right)\times S$ must be satisfied. Combining the above three constraints, the relationship among *D*, *P*, *S*, and $N_{c}$ can be expressed as:

(8) $$\left\{\begin{array}{c} \left(P-1\right)\times D\geq \left(N_{c}-1\right)\times S\\ \begin{array}{c} D\leq S\\ P\geq N_{c} \end{array} \end{array}\right.$$

For example, if the number of ports in memory block $B_{0}$ is 3, then the data range that can be represented by $p_{0}$, $p_{1}$, and $p_{2}$ is {x+0×D,x+1×D,x+2×D}. To cover the data range required by the convolution kernels, {x+0×S,x+1×S,x+2×S}, and given $N_{c}=3$, S=2, and P=3, the value of *D* can be uniquely determined as D=2.

**Multi-Dimensional Data Access Addressing Algorithm:** The previous section introduced the physical parameters of the on-chip buffer, such as the number of memory blocks and the number of ports. This section discusses the address mapping of data stored on the chip. As shown in Figure 4b, the memory block index $B_{i}$ is calculated according to the row index of the given activation *r*:

(9) $$B_{i}=\left\lceil \frac{r}{S}\right\rceil$$

For example, in Figure 4b, the input feature maps represented by green and yellow are both stored in memory block $B_{0}$.

Each memory block is divided into multiple logical groups according to the parameter *D*, such as the groups $G_{0}$ to $G_{3}$ shown in Figure 4b. The capacity of each logical group is P×D. Therefore, the group index in the on-chip cache can be determined based on the column index of the feature map’s logical address:

(10) $$G_{i}=\left\lceil \frac{c}{P\times D}\right\rceil +\left(r\%S\right)\times \left\lceil \frac{W_{win}}{P\times D}\right\rceil$$

and the port index $p_{i}$:

(11) $$p_{i}=\left\lfloor \frac{c\%(L\times S)}{S}\right\rfloor$$

Finally, the physical address of the data in each port is determined by:

(12) $$Addr=G_{i}\times D+\left[c\%\left(P\times D\right)\right]\%D$$

**Figure 5:**
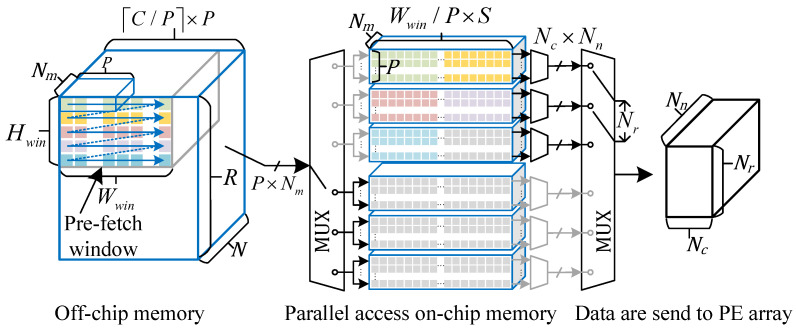
Activations are packed and aligned to improve the memory access efficiency.

**Efficient Memory Access Design for Binary Activation.** According to Equation (8), the relationship between port width *P* and degree of parallelism $N_{c}$ can be determined. Increasing port width *P* often implies increased resource consumption. However, moderately increasing *P* and performing multi-pixel packing on the input feature map can enhance the efficiency of off-chip data access. As shown in Figure 5, this work packs $N_{m}$ activations along the channel dimension with *P* binary activations along the horizontal dimension, forming a compact storage structure. Furthermore, the feature map is aligned along the horizontal direction with *P* pixels to ensure data access continuity, that is, the feature map width is expanded to ⌈C/P⌉×P as illustrated in Figure 5. In terms of accelerator hardware design, the corresponding off-chip activation access unit is configured with a port width of $P\times N_{m}$, and off-chip activations are read in the order of increasing addresses, as shown in Figure 5 to take advantage of the burst transfer capability of off-chip memory. Finally, based on Equation (8), the value of *P* is selected as 8 to achieve the goal of simultaneously fetching eight pixels along the horizontal direction in a single access. Moreover, the input feature map is divided into several pre-fetch windows, each of size $H_{win}\times W_{win}\times N$, and the group number of pre-fetch windows along the row and column direction equals $G_{l}^{r}$ and $G_{l}^{c}$, respectively. The data within each pre-fetch window is loaded in serial, and the on-chip buffer unit employs a ping-pong buffer design to overlap data transfer time with convolution computation time.

### 4.2. Computation Pipeline Design

**Figure 6:**
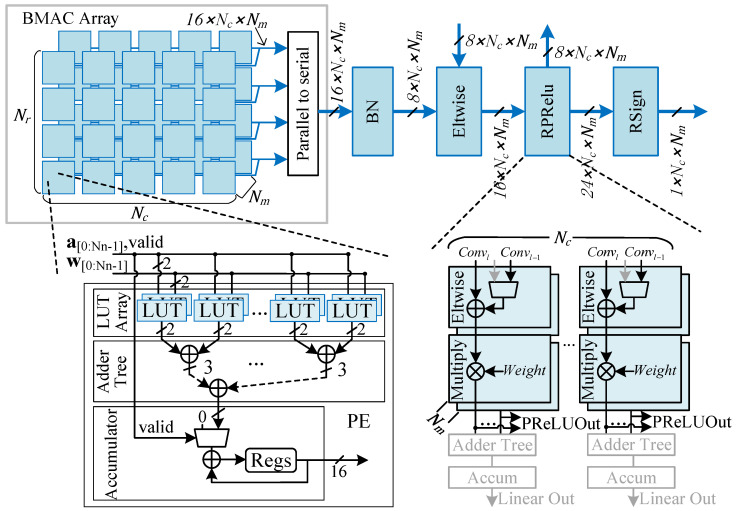
Hardware architecture of the computation pipeline.

As shown in Figure 6, the computation pipeline of the binary neural network accelerator is primarily composed of a BMAC computation array followed by subsequent computation units such as Batch Normalization (BN), shortcut connections, and PReLU. The BMAC array is a three-dimensional computation matrix consisting of numerous Processing Elements (PEs), which correspond to the parallelism in the height, width, and channel dimensions of the convolutional output feature maps. Each PE performs parallel binary inner product operations on $N_{n}$ input binary activations and weights, resulting in a total BMAC parallelism of $N_{r}\times N_{c}\times N_{m}\times N_{n}$.

A detailed view of a single PE is also illustrated in Figure 6. Unlike traditional binary convolution accelerators that use XNOR and bit-count operations as basic computations, the proposed design first computes the results of XNOR and corresponding bit-count operations directly through Look-Up Tables (LUTs). These results are then processed through an adder tree and an accumulator to complete the final inner product computation. This approach improves computational density, making more efficient use of LUT resources. Additionally, the design takes advantage of the characteristic that convolutional operations produce $N_{r}\times N_{c}\times N_{m}$ parallel convolution results every $k\times k^{\prime }\times N/N_{n}$ clock cycle. A parallel-to-serial conversion unit is implemented to reduce the hardware resources required for subsequent operations.

The PReLU unit shares DSP resources with the 1 × 1 convolution to perform 8-bit fixed-point convolution operations for the last three layers of the detection head. This multifunctional unit has parallelism in two directions, $N_{m}\times N_{c}$. When configured for PReLU operations, the data flow is represented by black lines in the figure: the convolutional output of the current layer is added to the output of the previous layer and then multiplied by the corresponding coefficient based on the sign of the sum. For the 1 × 1 convolution, the data flow is represented by gray lines. Input activations enter through the conv1 port and are multiplied by the corresponding weights, with the results accumulated to produce the final convolution output. Although only two degrees of parallelism are provided for fixed-point convolution, the computational workload of the detection head is relatively low, making the achieved speed acceptable.

On-chip intermediate data in neural network computations are represented with higher bit-width formats. For example, to prevent data overflow when convolution kernel size is large, the convolution outputs are represented using 16-bit fixed-point numbers. After BN, the data are quantized to 8-bit fixed-point numbers to balance the trade-off between computational precision and resource consumption during the subsequent shortcut operations. In the subsequent PReLU computation, the 16-bit intermediate data are multiplied by the corresponding weights producing 24-bit fixed-point results. These 24-bit results are then divided into two parts: one part is quantized to 8 bits and stored in off-chip memory for residual connections with the next layer, while the other part takes the sign bit to generate the binary input for the next layer.

### 4.3. Hardware Efficient Post-Processing Unit

**Figure 7:**
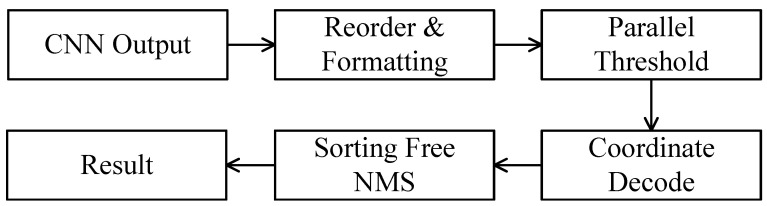
Work flow of the post-processing.

This chapter presents a hardware post-processing acceleration unit featuring low resource consumption and low latency. The proposed post-processing acceleration design is shown in Figure 7, which consists mainly of a data reorder unit, a parallel threshold unit, a confidence score decode unit, a coordinate decode unit and an NMS unit. We primarily improve the performance of post-processing through two key aspects: First, a parallel thresholding circuit is designed to filter out redundant candidate boxes according to the raw data from the BNN output, which reduces the workload in subsequent computations. Then, a hardware-friendly and sorting-free NMS algorithm and its FPGA implementation are proposed. Compared to the fully parallel pipelined NMS design proposed in [17], this design achieves a balance between resource consumption and computational latency.

**Efficient threshold unit design.** In order to reduce the computational overhead of subsequent decode and NMS operations, a parallel threshold unit is designed to filter out anchor boxes with low confidence and classification scores.

**Figure 8:**
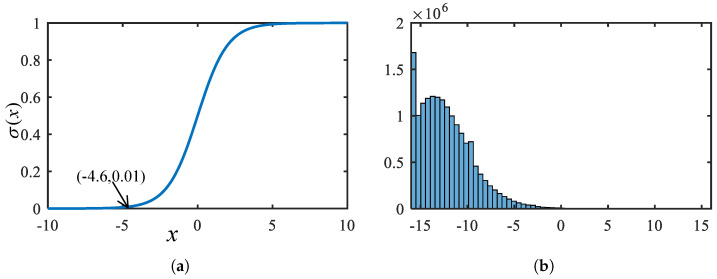
Sigmoid function and the distribution of BNN output: (**a**) Sigmoid function is used to decode the confidence information and 0.01 is set as a threshold for valid anchor boxes. (**b**) The distribution of the $t_{c}$ in SSDD dataset, where only 1.47% $t_{c}$ is larger than $\sigma^{-1}\left(0.01\right)$.

The confidence score of a candidate bounding box is obtained by decoding the neural network output using the sigmoid function, i.e., $\sigma (t_{c})$. We collect inference results on 1000 images from the SSDD dataset and analyze the distribution of the confidence scores. It is found that even with a very low confidence threshold, a large number of candidate boxes can still be filtered out. For example, when the threshold is set to 0.01, the corresponding input $t_{c}$ can be derived using the inverse sigmoid function, resulting in $t_{c}=-4.6$, as shown in Figure 8a. According to the distribution of predicted confidence scores on SSDD shown in Figure 8b, only 1.47% of the $t_{c}$ values are greater than −4.6. Therefore, applying the threshold operation earlier in the post processing pipeline can effectively reduce the computational load of subsequent processing steps.

Therefore, an efficient thresholding unit can filter out redundant candidate boxes and reduce the workload of subsequent decode and NMS operations. Due to the large number of anchor boxes, the threshold operation is most likely to become the bottleneck of the post processing unit. Therefore, to reduce latency, a parallel threshold unit is designed, where the hardware-intensive sigmoid function is replaced. Sigmoid function is nonlinear, current approaches include invoking a floating-point IP core [16] or approximating it using piecewise functions [27]. However, these methods typically lead to high hardware consumption when parallelism increases. To address this challenge, we calculate the convolution result corresponding to the threshold $T_{c}$ using

(13) $$\sigma^{-1}\left(T_{c}\right)=\ln\left(\frac{T_{c}}{1-T_{c}}\right).$$

**Figure 9:**
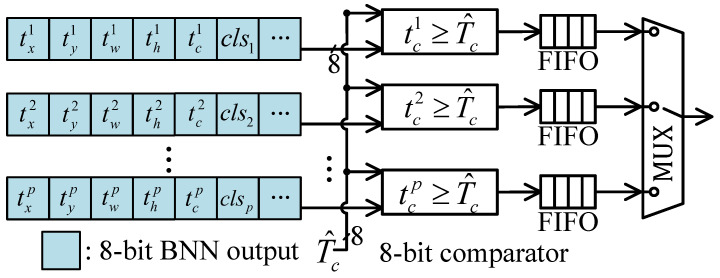
The sigmoid and threshold function is implemented as 8-bit fixed-point comparator.

Then, the candidate boxes with $t_{c}\leq \;sigma^{-1}\left(T_{c}\right)$ are filtered out. Considering that the convolution outputs are 8-bit quantized, the threshold can be further quantized by ${\hat{T}}_{c}=sigma^{-1}\left(T_{c}\right)\times 2^{fl}$. Therefore, the confidence threshold unit can be implemented by a series of 8-bit fixed-point comparators, as shown in Figure 9. The parallel threshold unit takes 8-bit fixed-point anchor boxes as inputs, and pushes the anchor into FIFO when $t_{c}\geq {\hat{T}}_{c}$. These FIFO connect the threshold unit and subsequent coordinate and confidence decode unit.

#### 4.3.1. Coordinate Calculation

The location of a prediction box is represented by the coordinates of two diagonal points, i.e., $\left(x_{1},y_{1}\right)$ and $\left(x_{2},y_{2}\right)$:

(14) $$\begin{array}{cc} x_{1} & =(b_{x}-0.5b_{w})\times scale\\ y_{1} & =(b_{y}-0.5b_{h})\times scale\\ x_{2} & =(b_{x}+0.5b_{w})\times scale\\ y_{2} & =(b_{y}+0.5b_{h})\times scale \end{array}$$

The scale denote the factor that maps the predicted boxes obtained at different scales back to the original image size. The $\left(b_{x},b_{y}\right)$ denote the coordinates of the bounding box [28], and $b_{w}$, $b_{h}$ denote the width and height of the bounding box, respectively. They are calculated by

(15) $$\begin{array}{cc} b_{x} & =\sigma \left(t_{x}\right)+c_{x}\\ b_{y} & =\sigma \left(t_{y}\right)+c_{y}\\ b_{w} & =p_{w}e^{t_{w}}\\ b_{h} & =p_{h}e^{t_{h}} \end{array},$$

where σ denotes the sigmoid function. The network predicts four coordinates for each bounding box, i.e., $t_{x}$, $t_{y}$, $t_{w}$ and $t_{h}$. The $\left(c_{x},c_{y}\right)$ denotes the offset from the top left corner of the image, and $p_{w}$ and $p_{h}$ represent the width and height of the anchor box, respectively.

**Figure 10:**
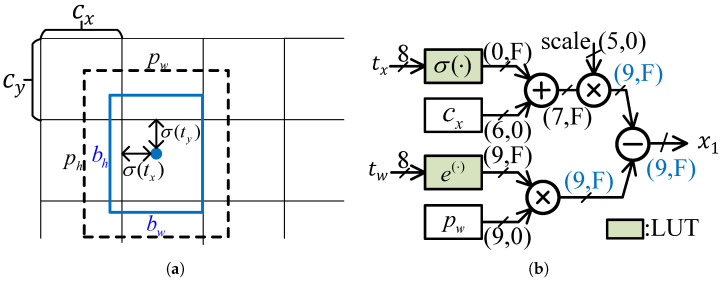
Coordinate decode algorithm and its hardware implementation: (**a**) Coordinate decode algorithm. (**b**) Hardware design of the coordinate decode unit.

Since $t_{x}$, $t_{y}$, $t_{w}$ and $t_{h}$ are 8-bit fixed-point data, their sigmoid and exponential results are calculated using the look-up table with low hardware costs. Moreover, the intermediate data of the coordinate decode stage are represented by fixed-point numbers, and are denoted as (I,F), where *I* and *F* denote the length of the integer and fractional bits. As shown in Figure 10, the output of sigmoid ranges between 0 and 1, so the integer length I=0. Moreover, the range of $c_{x}$ is between 0 and 52, so the integer length of $c_{x}$ equals $\lceil log_{2}^{52}\rceil =6$. Similarly, all data widths can be calculated according to their data range, and they are shown in Figure 10. Compared with floating point coordinate data, our proposed method balances hardware resources and precision by adjusting the parameter *F*. The correlation between *F* and accuracy and hardware consumption is illustrated in Section 5.3.

#### 4.3.2. Sorting-Free NMS Unit

After decoding the coordinates and confidence scores, the NMS is used to filter out redundant bounding boxes. A hardware-friendly NMS algorithm was proposed by [16], which omits confidence sorting to achieve low detection latency. However, that work consumed over 430 DSP blocks, which hinders implementation on resource-constrained edge devices. To achieve a balance between hardware resource consumption and performance, we propose an NMS algorithm and its implementation on FPGA.

The proposed algorithm is shown in Algorithm 1, which has multiple threads to accelerate the NMS process. Each thread maintains a local memory block denoted as $BOX_{sel}$ to store the selected candidate boxes, and $BOX_{num}$ is used to record the number of selected boxes. Whenever an input candidate box enters the NMS module, each thread calculates the IoU and compares scores with the candidate boxes stored in its local memory blocks. If the IoU is above the threshold and the confidence of the input box is larger than that of the selected box, the selected box will be replaced. The input box is appended to the selected boxes when the IoU is below the threshold. Furthermore, to ensure a balanced workload across threads, each input box is preferentially assigned to the $BOX_{sel}$ with the minimum $BOX_{num}$, which is reflected in lines 13 and 24 of the code. The proposed NMS hardware unit is mainly composed of a selected box buffer and an IoU calculation unit. The IoU calculation unit takes fixed-point coordinate data from the previous step. To avoid the hardware-expensive division operation, the IoU computation and comparison are replaced with

(16) $$\left|b_{s}^{t}\cap b_{i}|>\tau \;\cdot \;|b_{s}^{t}\cup b_{i}\right|$$

where the inequality holds when the IoU exceeds the threshold.

**Algorithm 1:** NMS implementation

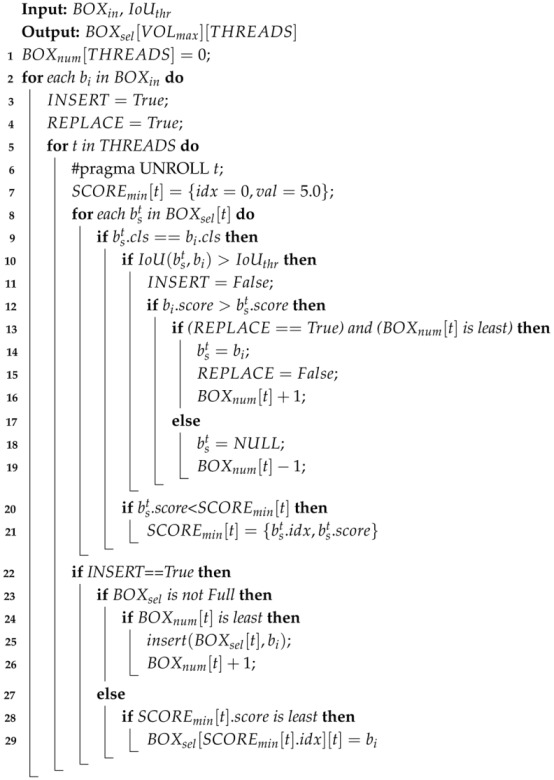



## 5. System Implementation and Performance Evaluation

### 5.1. Experimental Setup

To validate the feasibility of the proposed method, this work employs the PyTorch framework for training and validating the neural network algorithm. The hardware specifications of the algorithm server include: an Intel i9-9900k CPU, 64 GB of RAM, and an NVIDIA 4090 GPU with 24 GB of VRAM. The software environment consists of: Ubuntu 20.04 as the operating system, NVIDIA 535.183 GPU drivers, CUDA version 11.6, and PyTorch version 2.0. The backbone network was designed and initially trained on the ImageNet ILSVRC12 dataset [29]. Subsequently, the pre-trained backbone network was integrated into the proposed ship target detection model, and training was conducted using MM detection 2.26 on the SSDD [30], AIR-SARShip2.0 [31], and SAR-Ship [32] datasets for 200, 600, and 100 epochs, respectively. During training, the image resolution for SSDD and AIR-SARShip2.0 was set to 416×416. Due to the smaller image resolution in the SAR-Ship dataset, the resolution was set to 256×256 for training on this dataset. For the target detection network training, the batch size was set to 16, and the Adam optimizer [32] was used with an initial learning rate of 0.0001. Finally, the BRECQ quantization algorithm was applied to quantize floating-point operations in the network model.

Subsequently, the proposed accelerator was deployed and tested on FPGA devices. The FPGA compilation server’s hardware includes: an AMD Ryzen 9 7950x CPU and 64 GB of RAM. The system runs on Ubuntu 20.04, with Vitis, Vivado, and Petalinux versions 2022.1. First, the design files were compiled into RTL files using Vitis HLS and exported as IP cores. The accelerator IP was then imported using Block Design, followed by synthesis and implementation. The accelerator was deployed on a custom development board featuring the XC7VX690T device. The operating frequency of the proposed accelerator is obtained from the Vivado implementation results and is closely related to the resource utilization on the FPGA device. The latency of each convolutional layer is measured using a timer from the standard C language library. Since the convolution layers are executed sequentially, the timer is started at the beginning of each layer’s computation and stopped upon completion, allowing the inference latency to be recorded. The power consumption of the FPGA development board is measured using an external power meter connected to the board, while the power consumption of the FPGA chip itself is estimated using the Vivado Power Analyzer 2022.1 tool.

### 5.2. Resource and Power Evaluation

**Figure 11:**
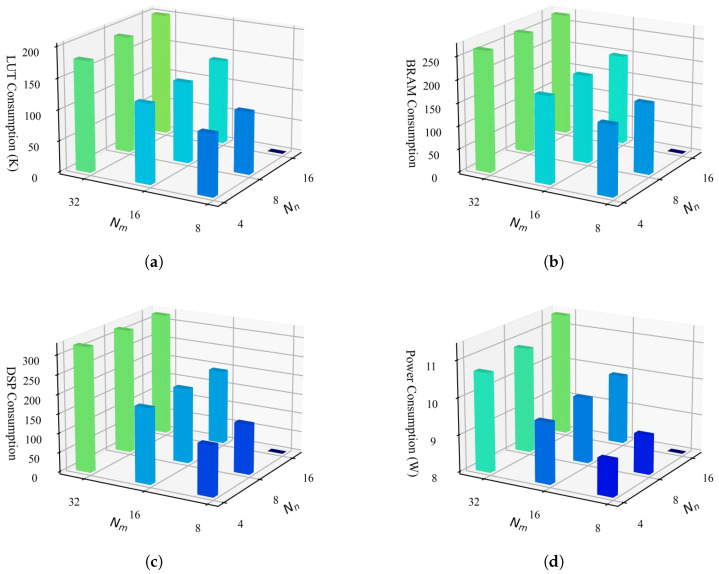
Resource and power consumption estimation on XC7VX690T device. (**a**) Logic resource. (**b**) Block RAMs. (**c**) DSP block. (**d**) FPGA device power consumption. Bars of different heights are highlighted in different colors for better visual distinction.

The resource utilization and power consumption are strongly influenced by the parallelism configuration of the accelerator, specifically the parameters $N_{r}$, $N_{c}$, $N_{m}$, and $N_{n}$. The correlation between hardware consumption and parameters $N_{m}$ and $N_{n}$ is shown in Figure 11, where, for clarity, $N_{r}$ and $N_{c}$ are configured as 7 and 4, respectively. Due to limitation in on-chip buffer design, the parameter $N_{m}$ must be greater than or equal to $N_{n}$; therefore, configurations where $N_{m}=8$ and $N_{n}=16$ are not included in the recorded results. Specifically, the LUT consumption is measured by

(17) $$C_{LUT}=c_{1}\times N_{r}\times N_{c}\times N_{m}\times N_{n}+c_{2},$$

where $c_{1}\times N_{r}\times N_{c}\times N_{m}\times N_{n}$ accounts for the logic resource consumed by the BMAC array, which primarily relies on LUT resource. The parameter $c_{2}$ measured the LUT consumed by the rest of modules.

The on-chip BRAMs are primarily consumed by the on-chip data buffers and the FIFOs that connect different accelerator kernels. The BRAM utilization is measured by

(18) $$C_{BRAM}=c_{3}\times N_{r}\times N_{c}\times N_{m}+c_{4}\times N_{m}\times N_{n}+c_{5}\times N_{c}\times N_{m}+c_{6},$$

where $c_{3}\times N_{r}\times N_{c}\times N_{m}$, $c_{4}\times N_{m}\times N_{n}$ and $c_{5}\times N_{c}\times N_{m}$ represent the BRAM consumed by the feature buffer, weight buffer, and output buffer, respectively. According to this model, BRAM utilization is primarily determined by the buffer volumes and the port widths of the buffers. As shown in Figure 11b, BRAM consumption increases significantly with larger $N_{m}$, whereas the influence of $N_{n}$ is relatively minor. This is because increasing $N_{n}$ only affects the size of the weight buffer, which requires minimal capacity due to the storage of binary weights.

The DSP resources are primarily utilized for executing PReLU and BN operations, and their consumption is mainly correlated with the product of $N_{m}$ and $N_{c}$, expressed as

(19) $$C_{DSP}=c_{7}\times N_{m}\times N_{c}+c_{8}$$

Based on the above analysis, the hardware resource consumption and power consumption increase significantly with the rise of $N_{m}$, while the impact of $N_{n}$ on hardware consumption is relatively minor. Therefore, we maximize $N_{n}$ to enhance the overall parallelism of the accelerator while incurring only marginal overhead in resource consumption. Through our design space exploration, we determined the optimal parameters as $N_{r}=7$, $N_{c}=4$, $N_{m}=32$, and $N_{n}=16$, achieving the maximum parallelism supported by the Xilinx XC7VX690T FPGA device.

### 5.3. Performance Evaluation

Our proposed BNN accelerator has $N_{r}\times N_{c}\times N_{m}$ CUs, and each CU performs $N_{n}$ BMAC operations in parallel. Therefore, the peak computational power provided by the BMAC array equates to

(20) $$C_{p}=2\times N_{r}\times N_{c}\times N_{m}\times N_{n}\times Freq.$$

The theoretical execution time of each layer equals the fraction of the computational workload and the peak computational power, i.e., $t_{e}=\#OP/C_{p}$, where the #OP denotes the computational workload and it equals to

(21) $$\#OP=2\times R^{\prime }\times C^{\prime }\times M\times k\times k^{\prime }\times N$$

**Figure 12:**
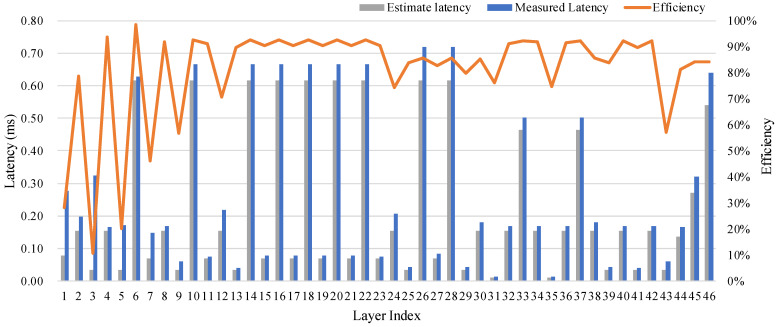
Theoretical and actual execution time of each layer of the HE-BiDet algorithm on the SSDD data set.

The execution efficiency is defined as the ratio of the measured computational power to peak computational power [26], i.e., $E=C_{m}/C_{p}$. The measured throughput is given by $C_{m}=\#OP/t_{m}$, where $t_{m}$ denotes the measured execution time of each layer. The theoretical and measured execution time and BMAC efficiency are shown in Figure 12. The computational efficiency for the first few layers is relatively low, and they are 1 × 1 convolutional layers. Since the input feature map of those layers is large, and their computational workload is relatively low, the throughput of those layers is memory bounded. However, the execution time of these layers is relatively short, so their impact on the overall system latency is minimal.

**Figure 13:**
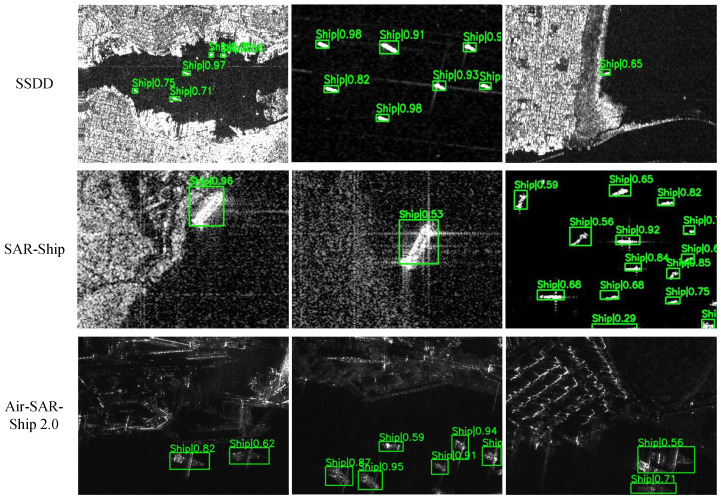
Visualization of the proposed HE-BiDet on three datasets.

The resource consumption and computational latency of both the neural network inference module and post-processing module in the accelerator are presented in Table 1. Since BMAC operations constitute the primary computations in binary neural networks, approximately 90% of the accelerator’s hardware resources are allocated to accelerate these BMAC operations. In contrast, the post-processing stage exhibits significantly reduced resource requirements and computational latency. This optimization is achieved through two key design strategies: (1) most nonlinear operations are implemented via lookup tables, and (2) the ship detection task only involves a single object category (ships), which substantially decreases the computational complexity of post-processing. Figure 13 presents visualized results of our proposed HE-BiDet accelerator across three distinct datasets. The visualization demonstrates robust detection capability under challenging conditions: (1) significant scale variations in ships (ranging from small to large vessels), (2) substantial clutter interference in surrounding waters, and (3) complex near-shore scenarios contaminated by coastal lines and artificial structures. Despite these challenges, our accelerator maintains accurate ship detection, and the consistent performance across diverse datasets validates the system’s generalization capability for maritime surveillance applications.

### 5.4. Comparison with State-of-the-Art Designs

Table 2 compares the work proposed in this paper with State-of-the-Art SAR image ship detection methods. The work [9] designed a ship detection model based on YOLOv5 and validated its accuracy on the SAR-SHIP dataset, achieving an AP50 of 78.74%. Furthermore, accelerator design and model deployment were completed on the Xilinx XCVU9P device, achieving an inference latency of 68.9 ms and a frame rate of 25.9 FPS. However, the model used in this work employed 16-bit floating point precision, which increased hardware resource consumption. Specifically, the work reported a requirement of approximately 5107 DSPs, necessitating the selection of the resource-rich XCVU9P chip. In contrast, our work achieves a 12.56% improvement in accuracy on the same dataset. Moreover, by utilizing binary representations for both model weights and activations, our approach requires only 322 DSPs, which is 6.3% of the DSPs used in [9]. Inference latency is reduced to 5.2 ms (7% of their latency), and the frame rate reaches 189.3 FPS, which is 7.3 times higher than that of [9]. The work [11] proposed a high-performance object detection accelerator for ship detection, achieving a frame rate of 636 FPS. This work achieved an accuracy of 93.3% on the SSDD dataset and was deployed on the Xilinx XC7VX690T device. Compared to this work, our proposed accelerator achieves a comparable accuracy of 92.7 % on the same data set. That work adopts low bit-width quantization, representing weights and activations with 4-bit and 3–6-bit data, respectively, and performing fixed-point convolutions using DSP resources. In contrast, in our proposed design, both activations and weights are represented by binary data, and the BMAC operations are implemented using logic resources. Due to the lower representational capacity of BNNs, a greater number of convolution layers is required to achieve comparable accuracy. Specifically, the model in [11] contains 28 convolution layers, whereas our proposed model includes 46 convolution layers. However, by leveraging binary activations and weights, the burden on off-chip memory access is significantly reduced, and the convolution computing unit is greatly simplified. As a result, higher computational parallelism can be achieved in our design.

In terms of inference speed, the high frame rate reported in [11] was achieved under a large batch size of 50. However, for practical deployment scenarios, such as edge devices, inference latency is more critical. Our work achieves an end-to-end inference latency of only 12.4 ms, which is 16.2% of the latency reported in [11], while the consumption of DSP is only 12.9% of theirs. The work in [33] adopted the traditional target detection method and achieved the detection latency of 16 ms. That work achieved a precision of 91.3% on their own dataset, where there are only 118 pieces of images. Compared with that work, our proposed work achieved a lower detection latency and our proposed work was evaluated on the public dataset with up to 43,819 [10] pieces of images.

We further compare our proposed accelerator with existing GPU-based methods. These include embedded GPUs such as the NVIDIA Jetson TX2 and desktop GPUs like the NVIDIA GeForce GTX 1050 Ti, as shown in Table 3. Our optimized HE-BiDet model achieves a good balance between accuracy, speed, and power efficiency. On the SAR-Ship dataset, it reaches 91.3% AP50 and 189.3 FPS, with a latency of only 5.2 ms. In comparison, the YOLOv5-based method [9] achieves 78.7% AP50, 8.0 FPS and 234.7 ms latency on the same dataset using GTX 1050 Ti. Our design consumes only 18.3 W, while the 1050 Ti requires 85 W, resulting in a 4.6× improvement in energy efficiency.

On the SSDD dataset, our model achieves 92.7% AP50, which is close to the 93.0% of YOLOv4 based method [34]. However, our model runs at 80.5 FPS, significantly higher than the 11.6 FPS of work [34] on the TX2. Power consumption is also comparable, with our accelerator using 18.3 W and the TX2 consuming 15 W. These results demonstrate that our accelerator provides high accuracy and speed, with low latency and efficient power consumption, making it suitable for real-time applications.

## 6. Conclusions

This work presents an optimized accelerator architecture for ship detection that achieves significant efficiency improvements through innovative 1-bit quantization of both weights and activations. The proposed design reduces the memory size by 18.9× and achieves 91.3% AP50 accuracy on the SAR-SHIP dataset, and 92.7% on the SSDD dataset, demonstrating robust capability in handling challenging SAR-specific conditions including wide scale variations. The accelerator features co-optimized BNN inference and post-processing stages, achieving real-time inference with 12.4 ms end-to-end latency. Due to the low resource consumption and scalable parallelism of our accelerator, it can be implemented on the resource-constrained space-grade FPGA devices. This broadens the application scenarios of our system, especially in spaceborne remote sensing and other resource limited environments, where high performance target detection is still required. Furthermore, a detailed comparison with State-of-the-Art designs in Section 5.4 confirms the superior performance, efficiency, and scalability of the proposed solution.

## Figures and Tables

**Table 1:** Performance and resource consumption of the accelerator.

	LUTs (K)	DSP	BRAM (36k)	Latency (ms) @10,647 Boxes
BNN Computation	184.9	299	261.5	12.12
Post Processing	5.3	23	10	0.04
Total	190	322	271.5	12.17

**Table 2:** Performance comparison between the SOTA FPGA based ship detection implementations using SAR imagery.

	IGARSS2024 [9]	TGRS2022 [11]	I2MTC2022 [33]	Ours	Ours	Ours
Model	YOLOv5	YOLOv2	Traditional	HE-BiDet	HE-BiDet	HE-BiDet
Model size (MB)	114.2	-	-	6.03	6.03	6.03
Dataset	SAR-SHIP	SSDD	-	SAR-SHIP	Air-SAR-SHIP	SSDD
Input Size	256 × 256	416 × 416	1000 × 1024	256 × 256	416 × 416	416 × 416
AP50	78.74	93.3	91.3	91.3	71.0	92.7
Data width (W/A)	FP16	4/3-6	8	1/1	1/1	1/1
FPGA Device	XCVU9P	XC7VX690T	XCKU115	XC7VX690T	XC7VX690T	XC7VX690T
Technology (nm)	16	16	16	28	28	28
LUTs (K)	-	196.9	77.1	190	190	190
DSP	5107	2496	120	322	322	322
BRAM 36K	-	319.5	970	271.5	271.5	271.5
Frequency (MHz)	-	250	-	180	180	180
Batch Size	1	50	1	1	1	1
Latency (ms)	68.9	76.68	16	5.2	12.4	12.4
FPS	25.9	636	62.5	189.3	80.5	80.5
Throughput (GOP/S)	-	-	-	3500.3	3929.7	3929.7
On-chip Power (W)	-	-	-	12.9	12.9	12.9
Platform Power (W)	36.8	-	-	18.3	18.3	18.3
GOPs/W	-	-	-	271.3	304.6	304.6

**Table 3:** Performance comparison between GPU-based works.

	Model	Dataset	AP50	Device	FPS	Batch Size	Latency (ms)	Power (W)
[9]	YOLOv5	SAR-Ship	78.7	1050 Ti	8.0	32	234.7	85
[34]	YOLOv4	SSDD	93.0	TX2	11.6	-	-	15
Ours	HE-BiDet	SAR-Ship	91.3	XC7VX690T	189.3	1	5.2	18.3
Ours	HE-BiDet	SSDD	92.7	XC7VX690T	80.5	1	12.4	18.3

## Data Availability

The original contributions presented in the study are included in the article, further inquiries can be directed to the corresponding author.

## Abbreviations

The following abbreviations are used in this manuscript:

CNN	Convolutional Neural Network
SAR	Synthetic Aperture Radar
BN	Batch normalization
FPS	Frame Per Second
NMS	Non-Maximum Suppression
PReLU	Parametric rectified linear unit
$N_{r}$	Parallelism of BMAC array in row direction
$N_{c}$	Parallelism of BMAC array in column direction
$N_{m}$	Parallelism of BMAC array in channel direction
$N_{n}$	Parallelism of within each PE
$B_{i}$	Bank index of the on-chip buffer
*P*	Port number of the on-chip buffer
$p_{i}$	Port index of the on-chip buffer
*S*	Stride of the convolution neural network
*K*	Kernel size of the convolution neural network
$R^{\prime }$	Number of rows of the output feature map
$C^{\prime }$	Number of columns of the output feature map
*M*	Number of channels of the output feature map
*N*	Number of channels of the input feature map
